# Effect of neck-specific exercises on trapezius muscle function in chronic whiplash-associated disorders: a longitudinal case–control study using ultrasound and speckle-tracking analyses

**DOI:** 10.1038/s41598-026-35963-y

**Published:** 2026-02-25

**Authors:** Gunnel Peterson, Erika Andersson, Margaretha Jönsson, Anneli Peolsson

**Affiliations:** 1 Centre for Clinical Research Sörmland, Uppsala University, Eskilstuna, Sweden; 2 Department of Health Medicine and Caring Sciences, Unit of Physiotherapy, Linköping University, Linköping, Sweden; 3 Independent researcher, Linköping, Sweden; 4 Independent researcher, Borås, Sweden; 5 Occupational and Environmental Medicine Centre, Department of Health Medicine and Caring Sciences, Unit of Clinical Medicine, Linköping University, Linköping, Sweden; 6 Centre for Medical Image Science and Visualisation, Linköping University,, Linköping, Sweden

**Keywords:** Whiplash injury, Ultrasonography, Neck muscles, Spine, Rehabilitation, Physical therapy, Diseases, Health care, Medical research, Signs and symptoms

## Abstract

Neck-specific exercises can improve the function of deep neck muscles in individuals with chronic whiplash-associated disorders (WAD), but it remains unclear whether these exercises also enhance the function of superficial neck muscles. The superficial trapezius muscle is frequently painful in WAD, yet effective conservative treatments are lacking. In total, 34 individuals with WAD and 34 healthy controls were recruited to assess upper trapezius function. Five participants from each group were excluded due to blurry ultrasound image or extreme outliers, resulting in 29 participants per group. Of the participants with WAD, 25 were reassessed after completing a 3-month neck-specific exercise program. Real-time, non-invasive ultrasound with speckle tracking was used to measure muscle deformation, enabling the detecting of subtle functional changes in the trapezius muscle that might not be captured by other methods. Participants rated neck disability, neck pain, and muscle fatigue at baseline and at 3-month follow-up (WAD group only). A significant group difference in trapezius deformation was observed at baseline (F[1,55] = 5.6, *p* = 0.022, ηp² = 0.09), with larger deformation in the WAD group. After three months, no significant group difference remained (F[1,47] = 2.3, *p* = 0.137, ηp² = 0.05). However, no significant within-group changes in deformation were found in the WAD group. In contrast, neck disability, neck pain, and muscle fatigue improved significantly following the intervention indicating a clear clinical benefit of the exercises. These findings suggest improved deformation of trapezius through neck-specific exercises, but the non-significant within-group change in muscle deformation warrants cautious interpretation.

Trial registration number and date of registration: The protocol was registered before data collection started at Clinicaltrial.gov (Protocol ID: NCT03664934, first posted date 11/09/2018).

**Supplementary Information:**

The online version contains supplementary material available at 10.1038/s41598-026-35963-y.

## Introduction

Although whiplash injuries after car crash rarely require intensive care or hospitalisation^1^, 50% of those injured with whiplash-associated disorders (WAD) report persistent symptoms years after the accident^2–4^. The symptoms of, for example, pain, dizziness, headache and fatigue, have an impact on daily life^3,5^. Neck pain is a dominant symptom in chronic WAD^6^ and was described by Tournier et al. as a major factor in lower health-related quality of life five years after WAD^4^.

The neck muscles work in a complex manner and interact with vestibular-, eye- and arm- movement control systems^7^. The deep neck muscle layers maintain postural control, initiate movement^8,9^ and cooperate with more superficial muscles to facilitate actions such as moving the head and arms^7^. The function of this intricate motor control system can change in neck pain, leading to delayed activation of the deep muscles^10,11^. Superficial muscles on the other hand, such as the trapezius, can be overused when the activation pattern changes in response to pain^10–12^. This may explain why repeated arm lifting increases neck pain in WAD^13^. Dysfunction of muscular control of the cervical spine after WAD has also been reported^10,14–17^.

The superficial trapezius muscle has been extensively investigated in individuals with neck pain, including WAD, and compared with healthy controls^18–22^. Trapezius dysfunction has been characterised by a decreased ability to relax after arm movement^18^, higher muscle stiffness^19,20^, and changes in biochemical processes^21^. In contrast, Voerman et al. observed no significant differences in muscle activation in the upper trapezius evaluated with surface electromyography (EMG)^22^. EMG assess neuromuscular function, the interaction between nerves and muscles, but investigating deeper muscle layers requires invasive EMG, which is not preferable in clinical practice. In contrast, ultrasound generates sound waves, and tissue echoes create grayscale images at various depths. This method has been validated for musculoskeletal muscles^23,24^ and enables non-invasive identification of changes in both deep and superficial muscle layers^25,26^. Ultrasound with speckle-tracking analysis is used to measure muscle deformation (lengthening or shortening) and can be applied clinically for diagnostic purposes and to evaluate exercise interventions. Another approach is real-time ultrasound shear-wave elastography, which measure muscle stiffness. However, stiffness in the upper trapezius was not correlated with neck pain^27^ and no changes in elastic properties were observed in the upper trapezius^28^. Thus, shear-wave elastography may not be an appropriate diagnostic method for WAD.

Various treatments targeting the trapezius muscle to reduce neck pain have been established and investigated, for example, friction massage^29^, transcutaneous electrical nerve stimulation^30^, dry needling^31^, and myofascial release^32^. However, these methods have only yielded modest results in the short and medium term. Exercise and information are recommended^33^ in chronic WAD, but to date, there is no clear evidence whether one exercise type is more effective than another. However, a neck-specific exercise programme has shown promising results^34–36^. If neck pain is primarily caused by dysfunction in the deep muscle layers or by altered coordination of motor synergies involved in neck movement^37^, then treatment approaches other than targeting the local trapezius muscle are required. Investigating real-time trapezius function may provide insights into the effectiveness of exercise interventions. Compared with EMG, speckle-tracking offers the advantage of dynamically assessing internal muscle deformation. Preferably, such investigation should closely reflect daily activities to enhance their relevance and applicability for patients.

The aims of the study were: (a) to investigate mechanical muscle function in upper trapezius during active arm elevation in participants with chronic WAD compared with healthy controls and (b) to compare mechanical muscle function after three months of a neck-specific exercise programme for the WAD group with baseline data of healthy controls. We hypothesised that deformation in the upper trapezius would be larger in patients with WAD than in controls at baseline. Furthermore, we hypothesised that participation in a three-month neck-specific exercises programme would improve deep cervical muscle function, thereby facilitating more efficient load transfer to superficial muscles and reducing between-group differences in trapezius deformation.

## Method

### Design and setting

This was a longitudinal case–control study, investigating function in the upper trapezius of individuals with chronic WAD grade II (neck pain and musculoskeletal signs) or III (neck pain, musculoskeletal signs, plus neurological signs) before and after a neck-specific exercise programme compared with healthy individuals. The study has an explorative design with an internal randomisation for the exercise delivery method among participants with WAD. The participants were recruited between 4 October 2018 and 16 December 2021. The study was approved by the Regional Ethics Review Board in Linköping, Sweden (ref. 2016/135–31 and 2017/556–32), and was conducted in accordance with the Declaration of Helsinki. The protocol was registered before data collection started at Clinicaltrial.gov (Protocol ID: NCT03664934, first posted date 11/09/2018) and the study protocol has been published^38^.

### Participants

Individuals with chronic WAD grades II–III with neck symptoms within the first week after a whiplash injury in a 4-wheeled motor vehicle crash were eligible for inclusion. For additional inclusion and exclusion criteria, see Textbox 1.

**Textbox 1:** Inclusion and exclusion criteria.

Inclusion criteria
• Persistent neck pain emanating from a whiplash injury six months to five years prior to study entry
• Average neck pain last week ≥ 20 mm on a 0–100 mm visual analogue scale
• Neck disability index (NDI) ≥ 20% (0%=no disability, 100%= highest disability)
• Age 18–63 years
• Daily access to a computer, tablet, or smartphone and the internet
• Sufficient time and motivation to follow the treatment program
**Exclusion criteria**
• Signs of head injury
• Serious physical pathology
• Surgery on the cervical spine
• Generalized or more dominant pain elsewhere in the body
• Previous fractures or dislocation of the cervical spine
• Other illness or injury that may prevent full participation in the study and/or for which exercises are contraindicated
• Previous severe neck problems that resulted in sick leave for more than 1 month in the year before the current whiplash injury
• Lack of knowledge of the Swedish language
• Diagnosed severe mental illness
• Current alcohol and/or drug abuse

Inclusion criteria for healthy controls, matched for age and sex, were: no present or past neck pain; no trauma to the neck or head, including whiplash injury; no neck or low back pain; no rheumatologic or neurological disease; no generalised myalgia; and right-handedness.

### Recruitment

Participants were recruited through healthcare providers, newspaper reports, social media, and the university’s website. Interested individuals completed a brief survey located on the study’s website. A project team member (physiotherapist) then contacted them to conduct a telephone interview, verifying their medical history and eligibility for the study. Eligible participants were scheduled for a physical examination by a physiotherapist, which was the final step in the recruitment process. If they met all the criteria, an appointment for an ultrasound test was arranged.

Healthy participants were recruited continuously from friends, family, staff, and social media. A project team member (physiotherapist) conducted a telephone interview to confirm their eligibility as healthy controls, and they completed a questionnaire.

### Procedure

#### Ultrasound measurement

**Fig. 1:**
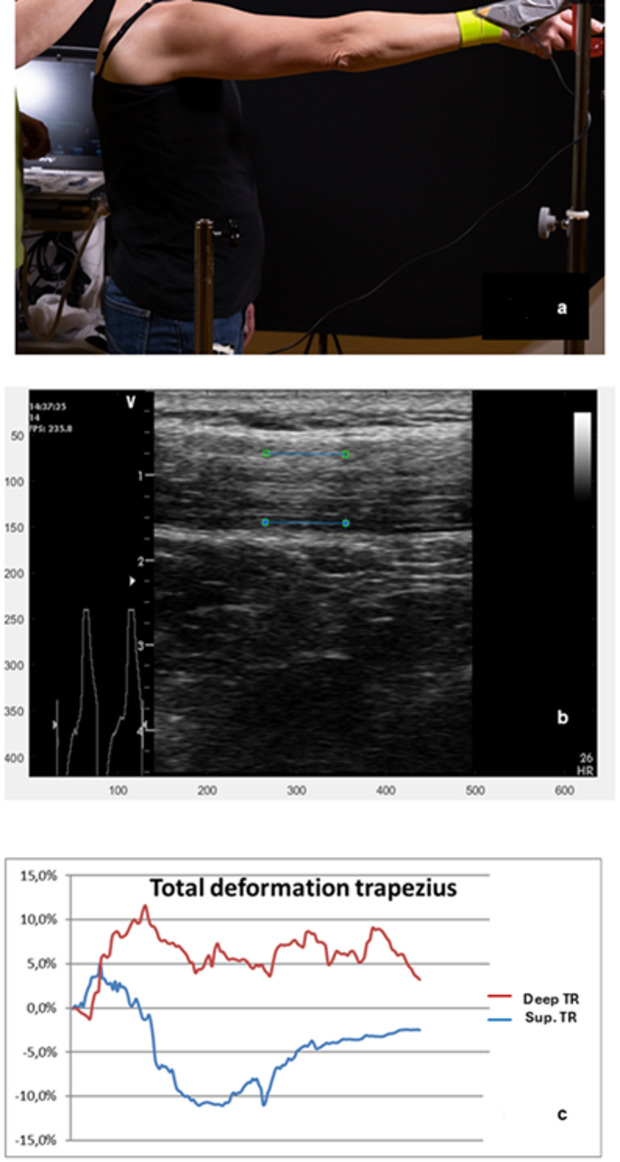
Ultrasound imaging and speckle tracking analysis. (**a**) Ultrasound measurement of the upper trapezius muscle. (**b**) The ultrasound image shows the longitudinal projection of region of interest (ROI), measuring the deformation (elongation and shortening) in the upper trapezius (TR). (**c**) The diagram illustrates muscle deformation sequences in the upper trapezius during one arm elevation to 90 degrees and back in the deep (deep TR) and superficial (Sup TR) part of the upper trapezius.

The right upper trapezius muscle was evaluated with a B-mode, 2-D ultrasound Vivid-I scanner (GE Healthcare, Horten, Norway) connected to a handheld 12 MHz linear transducer (38 mm) at 50 frames/s. The probe was positioned longitudinally, midway along the line from the acromion to the spine at vertebra C7, which is a standard position^39^ used previously for ultrasound recordings^40,41^. Each participant was asked to stand in a comfortable upright position with their feet behind a line marked on the floor (Fig. 1a). The right arm was raised to an adjustable horizontal bar fixed at 90º arm elevation, which was measured when the index finger touched the bar. Ten arm elevations were performed at a steady pace, following the beat of a metronome, set to 40 beats per minute. A custom-made contact switch was positioned in line with the participant’s right hip and at 90° arm elevation. Using the channel for the ECG system, a switch allowed data to be synchronised between arm movement and ultrasound measurements, which provided a cue for synchronising data between the ultrasonography and the starts and stops for each arm elevation. Participants held a weight of 0.5 kg (for females) or 1 kg (for males) in their right hand. They were instructed to move their arm smoothly, raising it on the first beat and lowering it on the next beat. To familiarise themselves with the test, each participant practiced using their left arm. The physiotherapists performing the ultrasound measurements were not blinded to group allocation. The tenth arm elevation was analysed because repeated arm elevations may increase pain and muscle fatigue in individuals with WAD but not for controls^13^, which could have an impact on muscle function. Based on our pre-study assessments, performing additional arm elevations would likely have been difficult for participants with WAD. The researcher performing post-process speckle-tracking analyses, including the placement of Regions of Interest (ROI), was blinded to group affiliation, as all ultrasound video sequences were coded.

Two 10 mm ROIs (Fig. 1b) were placed in the middle of the trapezius muscle (TR), aligned longitudinally with the muscle fibres, one in the superficial part 2 mm below the upper muscle border (superficial part of TR) and the second in the deep part of the muscle 2 mm above the lower muscle border (deep part of TR). Each ROI is indicated as a blue line with a square at each end. The muscle deformations for the superficial part (red line) and the deep part (blue line) of the upper trapezius in one participant are shown in Fig. 1c. Each line represents the changes observed in the ROI (deformation %) during the arm elevation. Muscle shortening is indicated by the area below zero (negative values), while muscle elongation is indicated by the area above zero (positive values). The sum of the negative and positive areas represents the total muscle deformation. When the line crosses 0%, the muscle deformation shifts from shortening to elongation, or vice versa.

#### Other measurements

Prior to undergoing ultrasound imaging, participants completed a baseline questionnaire which asked about their age, sex, average pain intensity over the past week, and current pain levels (VAS, 0 = no pain, 100 = worst imaginable pain)^42^ and neck disability (NDI, 0% = no disability, 100% = highest score for disability)^43^, WAD grade^44^, body mass index (BMI; weight (kg)/height in m²), neck muscle fatigue before and after the test (Borg CR-10 scale: 0 = no fatigue, 10 = extremely strong fatigue)^45^, and activity level (activity index: 1 = inactivity, 2 = low activity, 3 = moderate activity, 4 = high activity)^46^ (Table 1). Compliance with the exercise regimen was self-reported in the questionnaire at the 3-month follow-up in and categorised into five levels: Full compliance (80–100% of occasions); Predominantly (50–79% of occasions); Moderately (20–49% of occasions); Occasionally (< 20% of occasions); Rarely (a few times); Never.

**Table 1:** Baseline characteristics of participants with whiplash associated disorders (WAD) and healthy controls.

	WAD^a^ (*n* = 29)	Control (*n* = 29)	*P* value
Age, mean ± SD	40.7 (10.1)	41.6 (10.5)	0.74
Sex, female, n (%)	25 (83%)	25 (83%)	1.0
BMI^b^, mean ± SD	23.9 (3.8)	24.8 (3.3)	0.352
Months since injury, mean ± SD	27.5 (13.8)	NA	
WAD grade, grade III, n (%)	17 (59%)	NA	
Physical activity level^c^, n (%)			0.025
Inactive	1 (3%)	0 (0%)	
Low activity	11 (38%)	4 (14%)	
Moderate activity	9 (31%)	6 (21%)	
High activity	8 (28%)	19 (65%)	

^a^WAD: whiplash-associated disorders, grade III (neck pain, musculoskeletal signs, and neurological signs).

^b^BMI: body mass index; underweight: <18.5, normal weight: 18.5–24.9, overweight: 25–29.9, obesity: ≥30.

^c^Physical activity level during the previous 12 months (Inactivity = hardly any physical activity; Low activity = some physical activity (walk a few times/week); Moderate activity = walk several times a week and light exercise once a week; High activity = walk several times/week and moderate to vigorous exercises on a regular basis).

#### Neck-specific exercise programme

Participants with WAD were randomised to either neck-specific exercises with four visits to the physiotherapist over a 12-week period (NSEIT; *n* = 25) or neck-specific exercises with two visits per week to the physiotherapist (NSE; *n* = 4) in 12 weeks. Patients in both groups exercised over a 12-week period and were thereafter encouraged to continue training on their own two or three times a week, in accordance with the 2017 World Health Organization guidelines^47^ and to include NSEs in their training programmes. Due to the Covid-19 pandemic and limited capacity in the healthcare service, all patients recruited to the study after February 2020 were assigned to the NSEIT group (*n* = 14). The first exercises in the programme were targeted at facilitating and activating the deep neck muscle layers. These exercises were based on further studies of retraining neck muscles^48–50^. Thereafter, patients progressed to individualised endurance exercises within their symptom tolerance. Participants were encouraged to perform the exercises daily during the initial weeks. As their training progressed, the frequency of the exercises was gradually reduced (for details see^34)^.

The first session for the NSEIT group included a clinical examination and an introduction to the initial exercises. The follow-up sessions (weeks 2, 3, and 7) involved introducing new exercises, progressing existing exercises, and ensuring correct performance. Patients had access to an internet-based program, produced in-house by authors AP and GP for research purposes, which was available on a website. This program included information, photos, and videos of all exercises, with clear stepwise progression.

The patients in the NSE group received the same information and exercise programme but it was delivered by the physiotherapist at the clinic twice a week. They received the exercise programme in printed form. Initially, the exercises (involving activation of deep neck muscles) were also performed daily at home between physiotherapy visits.

All participants in both groups received the same information and the same neck-specific exercises, which have been clearly described previously^34^. The information covered whiplash injury, relevant musculoskeletal function, and neurophysiological and neurobiological mechanisms underlying chronic pain and neck pain relapse. The aim was to reassure patients and build confidence in performing the exercises.

#### Speckle tracking

An ROI was manually placed in the first frame of the video sequence for the muscle, making it possible to track the unique speckle pattern frame by frame through the video sequence. When the speckle pattern changes length with muscle activity, so does the length of the ROI. Elongation or shortening of the muscles, i.e. muscle deformation, was calculated as the percentage change from the original length of the ROI compared with rest, and was expressed as % deformation. The speckle-tracking methodology was based on an algorithm developed by Kanade-Lukas-Tomasi^51^, which was further enhanced with the methodology described by Farron et al.^52^. The speckle tracking methodology was implemented with an in-house software program written in Matlab 2013b^53^. The method is described in detail in our previous study^15^. The speckle tracking method has been validated for force measurements in musculoskeletal application^24,54,55^. The test-retest reliability is moderate to excellent (ICC 0.71 to 0.97, standard error of the mean 0.40 to 0.93) for muscle deformation^56^.

#### Statistics

All data were analysed using IBM SPSS Statistics for Windows (Version 29.0. IBM Corp, Armonk, NY, USA). Demographic characteristics of participants were compared between groups using the two-tailed unpaired Student t-test for parametric data; physical activity levels and neck muscle fatigue were analysed using the Mann–Whitney U-test and Pearson’s χ2 was used for categorical variables. Cohen’s d was used to estimate effect size for pain and disability and interpreted as *d* 0.2 < 0.5 = small effect, *d* 0.5 < 0.8 = medium effect and *d* > 0.8 = large effect^57^. Cohen’s *r* was used for non-parametric test and interpreted as *r* 0.1 < 0.3 = small effect, *r* 0.3 < 0.5 moderate effect and *r* ≥ 0.5 large effect^57^.

A convenience sample of 34 participants in each group was recruited. Post-process speckle tracking analyses (Fig. 1b) were performed, with a 10 mm ROI, positioned longitudinally to the muscle fibres. The ROI was manually placed in the first frame of the video sequence for the muscle.

Three mixed-design analyses of variance (ANOVA) with Bonferroni correction were used to evaluate: (1) between-subject factor of group and within-group factor of deformation at baseline (two groups, WAD and controls × two [superficial and deep] parts of the upper trapezius), adjusted by sex (male, female); (2) between-subject factor of group and within-group factor of deformation of the upper trapezius between the WAD group after the exercise intervention and the control group baseline data (two groups, WAD three months’ follow-up data and control baseline data × two [superficial and deep] parts of the upper trapezius) adjusted by sex; (3) the within-group effect in deformation in the upper trapezius after the exercise intervention in the WAD group (two timepoints, baseline, and at three-months’ follow-up × two [superficial and deep] part of the upper trapezius). In the present study, the two exercise groups (NSE and NSEIT) were analysed as one exercise group. The decision was based on findings from another randomised controlled trial, which reported equally favourable outcomes and no significant differences between the NSE and NSEIT groups in neck pain or disability^34^.

In mixed ANOVA analyses, Mauchly’s test was used to test the assumption of sphericity, and if this assumption was violated (*p* < 0.05) the Greenhouse–Geisser correction was used and the assumptions of equal variance were met (Levene’s test *p* > 0.05). Effect sizes, partial eta-squared (ηp2), were reported, with 0.01 indicating a small effect, 0.06 indicating an intermediate effect, and 0.14 indicating a large effect^57^. Bonferroni correction was applied to all multiple pairwise contrasts between the muscle levels. P-values ≤ 0.05 were considered to be statistically significant.

## Results

**Fig. 2:**
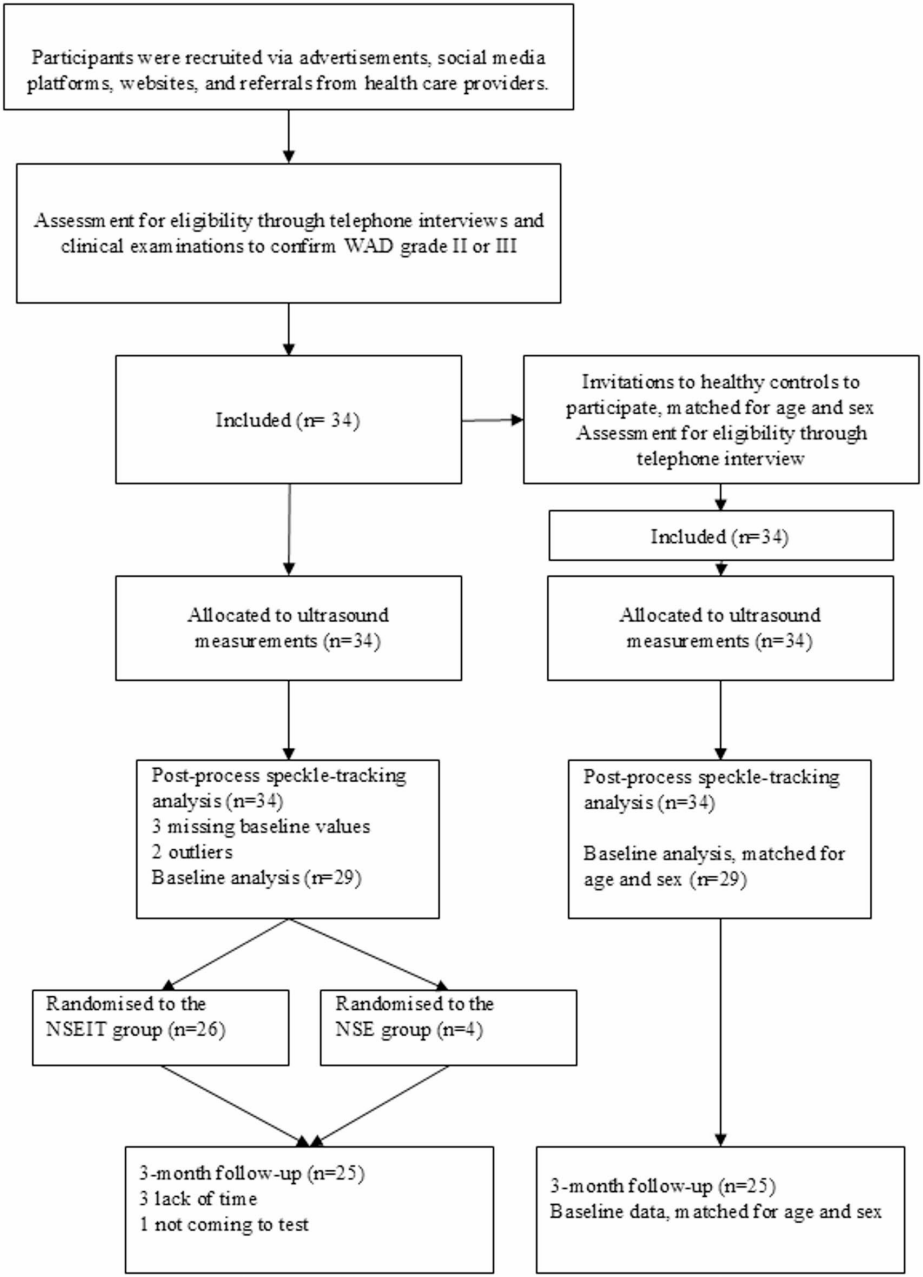
Flow chart.

In total, five participants from each group were excluded. The reasons were missing baseline data (blurry ultrasound image, three participants), or outliers at baseline, defined as values exceeding three standard deviations from the mean. Consequently, 29 participants with persistent WAD were included in the analyses (mean age 40.7 years (SD 10.1) and 29 healthy controls (mean age 41.6 years (SD 10.5), see baseline characteristics in Table 1. At baseline, there were no significant differences between the groups, except for a lower physical activity level in the WAD group (*p* = 0.025). Four participants in the WAD group were lost to follow-up at three months, giving 25 individuals from each group (3 months’ follow-up data were used from the WAD group and baseline data were used from the control group, matched for age and sex). No significant differences were found between dropouts and participants on any baseline variables (*p* > 0.302), except a near-significant difference in time since whiplash (dropouts: 14.5 months, SD 3.1; participants: 26.8 months, SD 14.5; *p* = 0.062). Individuals from the WAD group received three months of neck-specific exercises (Fig. 2). Full self-reported compliance with the exercise regimen was observed in 58.3% of participants (*n* = 14), while 29.2% (*n* = 7) reported compliance of 50–79% and 8,7% (*n* = 2) reported compliance of 20–49% during the 12-week intervention. Stratified analysis of exercise adherence is presented in Supplementary 1, Table S1. No adverse events were reported.

### Comparisons between the WAD and control groups at baseline

**Fig. 3:**
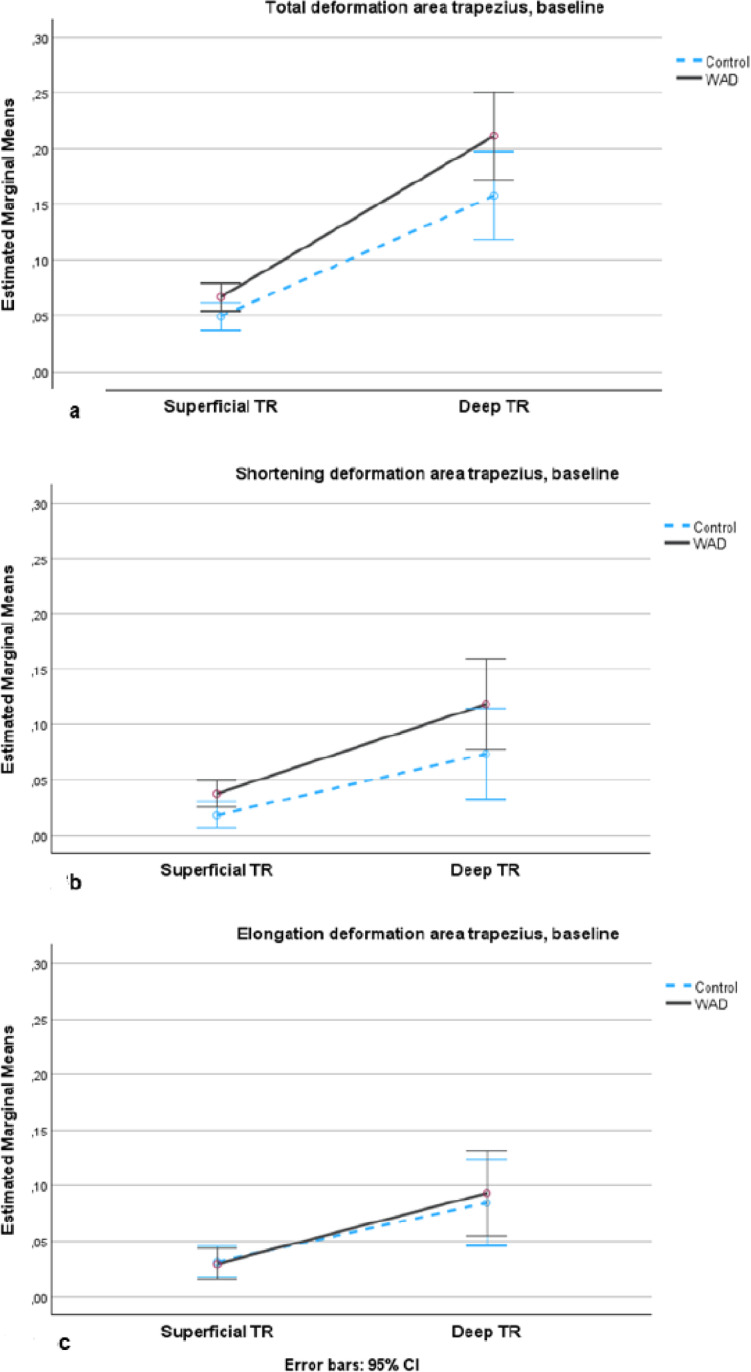
Deformation area in the upper trapezius in WAD and control groups at baseline. (**a**) Total deformation area represents the sum of elongations and shortenings of the muscles; (**b**) Deformation area shortening; (**c**) Deformation area elongation. Y-axis: the estimated marginal mean values (deformation area) with a 95% confidence interval (CI) in the upper trapezius muscle. X-axis: Superficial TR: superficial part of upper trapezius; Deep TR: deep part of upper trapezius are shown for the WAD (black line) and control groups (blue dotted line).

There was a significant main effect of deformation in the trapezius between groups (F[1,55] = 5.6,), *p* = 0.022, ηp2 = 0.09), estimated marginal mean EMM; 3.5 (95% CI; 0.5 to 6.6) with larger deformation area in both the superficial and deep part of the trapezius in the WAD group (Fig. 3a). There was a significant difference of deformation between the superficial and deep parts of the trapezius in both groups (F[1,55] = 8.1, *p* = 0.006, ηp2 = 0.13), EMM 12.6 (95% CI; 9.8 to 15.5), with larger deformation in the deep part of the trapezius. There were no differences between sex (F[1,55] = 0.5, mean *p* = 0.481, ηp2 = 0.009). The control group had significantly smaller shortening deformation in the trapezius (F[1,55] = 4.9, *p* = 0.032, ηp2 = 0.08), EMM 3.2 (95% CI 0.3 to 6.1) compared with the WAD group, but there were no differences between the groups in elongation deformation (F[1,55] = 0.6, *p* = 0.809, ηp2 = 0.001), EMM 0.3, (95% CI; −2.5 to 3.2) (Fig. 3b, c). There was no significant difference in test time between the WAD and control groups, *p* = 0.63. Deformation values and test times for both the WAD and control groups at baseline and at 3-months’ follow-up for the WAD group are presented in Table 2.

### Deformation area in the WAD group after three months of neck-specific exercise versus baseline data in the control group

**Fig. 4:**
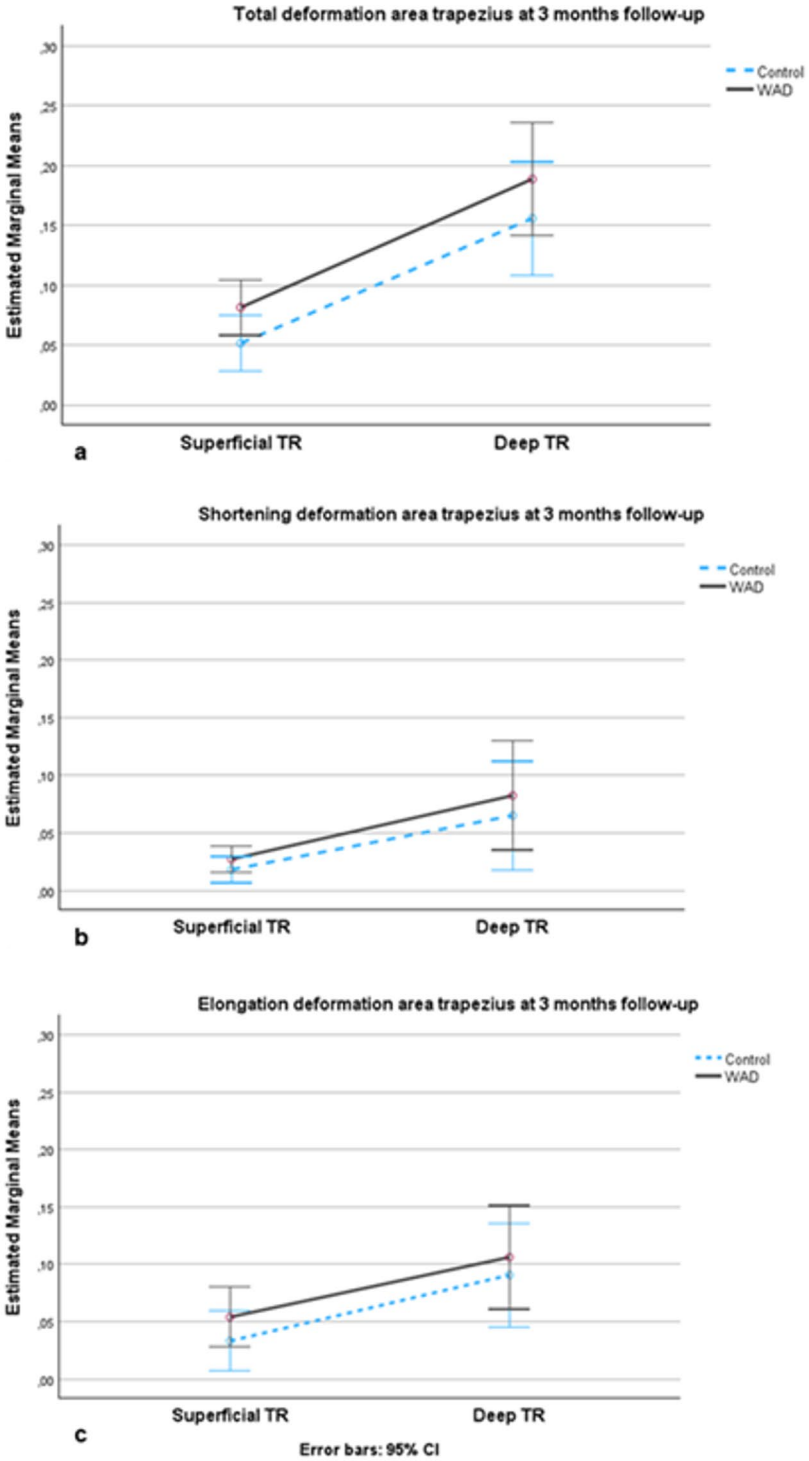
Comparisons of deformation area in the upper trapezius in the WAD group after three months of neck-specific exercise versus baseline data in the control group. (**a**) Total deformation area represents the sum of elongations and shortenings of the muscles; (**b**) Deformation area shortening; (**c**) Deformation area elongation. Y-axis: the estimated marginal mean values (deformation area) with a 95% confidence interval (CI) in the upper trapezius muscle. X-axis: Superficial TR: superficial part of upper trapezius; Deep TR: deep part of upper trapezius are shown for the WAD (black line) and control groups (blue dotted line).

There was no significant main effect of deformation between the groups after three months of neck-specific exercises in WAD compared with baseline data in the control group (F[1,47] = 2.3, *p* = 0.137, ηp2 = 0.05), EMM 3.1, (95% CI; −1.0 to 7.3). Accordingly, there were no differences between the groups in shortening (F[1,47] = 0.59, *p* = 0.445, ηp2 = 0.012), EMM 1.3, (95% CI; −2.1 to 4.8) or elongation (F[1,47] = 0.69, *p* = 0.411, ηp2 = 0.014) deformation, EMM 1.8, (95% CI; −2.5 to 6.2) (Fig. 4a-c).

### Within subject effects in the WAD group from baseline to 3 months’ follow-up

There were no significant within-subject effects between baseline and 3 months’ follow-up in the superficial (F[1,23] = 0.12, *p* = 0.728, ηp2 = 0.005) or in the deep part of the upper trapezius (F[1,23] = 0.35, *p* = 0.558, ηp2 = 0.015) in the WAD group.

**Table 2:** Muscle deformation area (expressed at % change in length) in the upper trapezius during the tenth arm elevation. Total area represents the sum of elongations and shortenings of the muscle, expressed as the mean and standard deviation for each group.

		WAD^a^	Control	WAD
		Baseline (*N* = 29)	Baseline (*N* = 29)	3 months (*N* = 25
**Test time**		3.10 (0.22)	3.13 (0.27)	3.11 (0.21)
**Total area** ^b^				
Whole group	Sup.TR^e^	6.7 (3.8)	5.0 (2.7)	8.1 (7.6)
	DeepTR^f^	21.1 (11.7)	15.8 (9.3)	18.9 (13.7)
Women	Sup.TR	6.6 (3.7)	4.9 (2.8)	8.4 (8.2)
	DeepTR	22.4 (11.7)	15.3 (9.7)	18.4 (12.4)
Men	Sup.TR	7.2 (4.7)	4.9 (1.1)	6.5 (2.4)
	DeepTR	14.9 (10.3)	18.9 (6.3)	22.1 (21.3)
**Shortening** ^c^				
Whole group	Sup.TR	3.8 (3.9)	1.8 (2.4)	2.8 (3.2)
	DeepTR	11.7 (12.4)	7.5 (9.7)	8.2 (13.7)
Women	Sup.TR	3.4 (3.3)	1.7 (2.4)	2.2 (3.0)
	DeepTR	13.1 (13.0)	7.9 (9.9)	7.2 (11.4)
Men	Sup.TR	5.5 (6.1)	2.3 (3.0)	5.7 (3.0)
	DeepTR	5.1 (6.5)	4.2 (8.2)	13.8 (24.0)
**Elongation** ^d^				
Whole group	Sup.TR	2.9 (4.1)	3.2 (3.2)	5.3 (8.6)
	DeepTR	9.4 (10.3)	8.4 (10.4)	10.7 (11.7)
Women	Sup.TR	3.2 (4.4)	3.3 (3.4)	6.2 (9.1)
	DeepTR	9.3 (10.3)	7.4 (10.0)	11.2 (11.9)
Men	Sup.TR	1.7 (2.5)	2.6 (2.0)	0.7 (1.4)
	DeepTR	9.7 (11.7)	14.7 (11.4)	8.2 (11.5)

WAD^a^: whiplash-associated disorders.

Total area^b^ = total deformation area; Shortening^c^ = shortening deformation area; Elongation^d^ = elongation deformation area.

Sup.TR^e^ = superficial part of the upper trapezius; DeepTR^f^ = deeper part of the upper trapezius.

There were significant improvements from baseline to three months’ follow-up in neck disability (NDI; mean − 8.2, SD 8.8, *d* = 0.86, *p* < 0 0.001), neck pain (VAS; mean − 16.6, SD 25.8, *d* = 0.66, *p* = 0.004), and neck muscle fatigue after tests (Borg CR-10; mean 1.5, IQR 0.0 to 3.3, *r* = 0.53, *p* = 0.009) (Table 3) in the WAD group. There was a statistically significant, moderate correlation between improvement in neck muscle fatigue and smaller deformation in the superficial part of the upper trapezius, *r* = 0.446*, *p* = 0.029, but no significant correlation between trapezius deformation and neck pain or disability (Supplementary file1, Table 2S).

**Table 3:** Outcomes in neck disability, pain and neck muscle fatigue for the WAD and control groups at baseline, and 3-months’ follow-up and change score for the WAD group.

	WAD		Control group (CG)	Baseline WAD vs. CG	With-in group change WAD	*P* value
	Baseline	3 months	Baseline	*P* value	3 months	
NDI	42.0 (13.7)	34.8 (15.1)	1.7 (1.6)	< 0.001	−8.2 (8.8)	< 0.001
VAS	42.3 (21.3)	24.6 (20.2)	0.03 ± 0.2	< 0.001	−16.6 (25.8)	0.004
Borg	4.0 (3.0 to 6.0)	3.0 (0.5–4.0)	0.0 (0.0–0.0)	< 0.001	−1.5 (0.0–3.3)	0.009

WAD^a^: whiplash-associated disorders.

NDI: Neck Disability Index 0–100% scale (0% = no disability to 100% = complete disability), mean (Standard Deviation).

VAS after test: visual analogue scale 0–100 mm (0 = no pain, 100 = worst imaginable pain), mean (Standard Deviation).

Borg CR 0–10 after test: neck muscle fatigue (0 = no fatigue, 10 = extremely strong fatigue), median and interquartile range.

## Discussion

The superficial trapezius muscle is often painful in WAD, yet treatments targeting the trapezius have shown only modest effects. Neck-specific exercises improve deep muscle function, but their impact on superficial muscles remains unclear. Ultrasound with speckle-tracking can assess trapezius deformation and may help evaluate the exercise programme. The present study showed larger deformation in both the superficial and deep parts of the upper trapezius in individuals with chronic WAD compared with healthy controls in an arm elevation task. More specifically, the shortening deformation was significantly larger in individuals with WAD but there were no differences in elongation deformation compared with the control group. After three months of a neck-specific exercise programme for the WAD group, there was no significant difference in deformation compared with the control group’s data. These findings indicate a possible alteration in trapezius muscle function following neck-specific exercise. However, the intermediate effect size and the absence of statistically significant within-group differences in muscle deformation warrant cautious interpretation.

Previous studies have demonstrated that ultrasound with speckle-tracking analyses can detect altered function in the deep ventral^15^ and dorsal^17^ neck muscles in chronic WAD. Moreover, three months of NSEIT or NSE improved deep neck muscle function^26^ and the interactions between deep and superficial ventral neck muscles during arm lifting^25^. In the present study, the NSE programme demonstrated that exercises aimed at activating and progressively enhancing endurance in the deep neck muscles^34^ also positively impacted upper trapezius function. There may be an association between improved deep neck muscles function after neck-specific exercises^25,26^ and the reduced deformation in the trapezius muscle. The stability of the cervical spine relies on support from surrounding neck muscles, and the deepest layers are particularly important for stability^9^. Individuals with WAD demonstrated greater activation of the trapezius during a repetitive upper limb task^58^, while reduced activation of deep neck muscles has been reported in those with neck pain^12^. These findings suggest a possible association between overactivation of superficial neck muscles and impaired deep neck muscle function.

However, there were no significant within-group differences in upper trapezius deformation from baseline to the three-month follow-up in the WAD group. Therefore, the NSEIT and NSE programmes were not sufficient to improve muscle deformation significantly in the upper trapezius. As shown in Fig. 4a-c, the mean deformation values in the WAD group are closer to those of healthy controls compared with baseline values (Fig. 3a-c), although a difference in deformation remains. These results suggest that other exercises or treatments are needed to complement NSEIT/NSE. The heterogeneity of symptoms observed in chronic WAD, along with factors such as work demands and structural variations in the cervical spine, may have influenced the results, as it is not possible to control for all confounding variables. Neck pain, disability, and neck muscle fatigue demonstrated significant improvement at the three-month follow-up in the WAD group, with effect sizes ranging from medium to large. Neck pain and neck muscle fatigue were measured immediately after the arm lifting test, indicating that an activity often used in daily life was less painful after three months of NSEIT/NSE.

The upper trapezius is often stiff and tender to palpation and touch in WAD and consequently, many treatment methods have been directed to the upper trapezius in neck pain. However, these methods only yielded small or very modest results^29–32^. These treatment methods may rely on earlier biological theories that proposed that pain leads to muscle spasm^59,60^ or higher activity in antagonist muscles^61^, or these treatments may be used because the trapezius muscle is easier to treat than deeper muscle layer. Further research and development of new treatment methods should be based on pathophysiological diagnoses, knowledge of pain neurological mechanisms, and the biopsychosocial aspects^62^ of chronic pain.

The trapezius muscle is relatively easy to investigate using methods such as ultrasound^19^, surface electromyography (EMG)^22^ microdialysis technique and pressure algometer^21^. The advantage of real-time ultrasound sequences with speckle-tracking analysis is that this non-invasive method provides information on deformation at different levels within a muscle or between muscle layers. In the present study, deformation was categorised into shortening and elongation. Although only EMG measures the neuromuscular activation of muscle fibres, the ultrasound method with speckle tracking analyses gives valuable information on mechanical muscle function (deformation). In contrast, shear-wave elastography appears less suitable as it has not detected differences in muscle stiffness in individuals with neck pain^27,28^. These findings underscore the potential of speckle-tracking as a sensitive tool for monitoring rehabilitation progress in patients with WAD and for advancing our understanding of muscle function, warranting further investigation in larger studies. Furthermore, the results demonstrated that individuals with WAD exhibited larger shortening deformation in the deep portion of the trapezius compared to controls, highlighting the need for targeted strategies that address this muscle´s deeper layer.

### Limitations

This study has some limitations. The aetiology of chronic WAD with pain persisting from six months to five years following whiplash injury is heterogeneous with high variations in muscle deformation between the participants, which may have affected the results. The study revealed significant baseline differences between the WAD and control groups, which diminished following a three-month neck-specific exercise programme in the WAD group. However, due to the non-significant findings in the within-group analyses, these results should be interpreted with caution. At baseline, there was a significantly lower physical activity level in the WAD group, a confounding factor that may have implication on results. However, low physical activity may be associated with pain, disability, and reduced neck muscle endurance, factors that have been shown to improve following the exercise program^63^. The physiotherapists performing the ultrasound measurements were not blinded, which could have introduced bias. However, measurements followed a strict protocol, and consistent probe positioning was required for clear video sequences. The researcher conducting the speckle-tracking analyses, including placement of Regions of Interest (ROI), was blinded to group affiliation, because all ultrasound video sequences were coded. We believe that the lack of blinding during data collection did not influence the interpretation of results, as the analyses were performed using coded speckle-tracking data.

The healthy control group was assessed only at baseline, without 3-month follow-up, which may have affected the findings. Including repeated measurements in the control group could provide a more comprehensive understanding of muscle function and should be considered in future research. The exercise programme did not include any exercises targeting the trapezius muscle that may impact the results. Despite that, the results in the present study could be a starting point for further evaluation of neck muscle impairments and exercise interventions in WAD grades II and III but further studies with a larger sample size are required to confirm the results. With only four men included, the findings are primarily generalizable to women and should be interpreted with caution for men. The short follow-up time is a limitation, as results in the long term are of great interest. However, earlier studies have shown sustained results in neck pain, disability and neck muscle endurance up to one year after three months of the NSEIT or NSE programme^34,63^. Part of the study was conducted during the COVID-19 pandemic, which restricted access to health care and may have influenced the intervention. Nevertheless, participants in the NSEIT group performed their exercises at home, and none contracted COVID-19. In addition, physiotherapy visits during the pandemic adhered to the study protocol and were consistent with those conducted before the pandemic. In addition, participants were recruited through healthcare providers, newspaper reports, social media, and the university’s website. Recruitment via the university may have resulted in a higher proportion of participants with advanced education, potentially limiting the generalisability of the results.

In conclusion, ultrasound measurement with speckle-tracking analysis indicated improved deformation of upper trapezius muscle function, along with improvement in neck pain, disability and neck muscle fatigue in individuals with chronic WAD after NSEIT/NSE.

However, the non-significant within-group results for WAD indicate that complementary interventions, such as muscle strengthening, relaxation exercises or manual therapy, may be required to effectively improve trapezius muscle function.

## Supplementary Information

Below is the link to the electronic supplementary material.

Supplementary Material 1

## Data Availability

The data generated during the current study are available from the corresponding author on reasonable request.
