# Supplementary material for: Effect of neck-specific exercises on trapezius muscle function in chronic whiplash-associated disorders: a longitudinal case–control study using ultrasound and speckle-tracking analyses
Source: Sci Rep. 2026 Feb 25;16:7725. doi: 10.1038/s41598-026-35963-y (PMC12949063; doi:10.1038/s41598-026-35963-y)
Supplement: Supplementary file 1 — Supplementary Material 1 [file 41598_2026_35963_MOESM1_ESM.docx]

Supplementary file 1

Table 1S. Stratified analysis of adherence to exercises, change score from baseline to 3 months follow-up in neck pain, neck disability, neck muscle fatigue and deformation area trapezius

|  |  |  |  |  |  |
| --- | --- | --- | --- | --- | --- |
|  | Complience to exercise > 80% | Complience to exercise 79 to 50 % | Complience to exercise 20 to 49 % | P value |  |
| **WAD patients** | 14 (58.3 %) | 7 (29.2%) | 2 (8.7 %) |  |  |
| **NDI** | 8 ( 17 to 5.5) | 10 (14 to -4) | 11 (11 to -16) | 0.845 |  |
| **VAS** | 16 (31.5 to 5) | 25 (38 to -16) | 1 (1 to 0) | 0.423 |  |
| **Borg** | 1.5 (3.2 to 0) | 0.5 (3.5 to -2) | 1.5 (.51 to -1) | 0.637 |  |
| **Deformation Sup.TR** | -1.2 (-3.5 to 2.1) | -0.6 (-1.8 to -0.05) | 3.5 (-7.2 to 3.6) | 0.895 |  |
| **Deformation DeepTR** | 6.6 (-1.6 to 11.8) | -10.9 (-16.9 to 1.5) | 22.6 (13.6 to 22.6) | 0.014 |  |
|  |  |  |  |  |  |

WAD: whiplash-associated disorders, n (%)

NDI: Neck Disability Index 0–100% (0% = no disability to 100 % = complete disability), median and interquartile range, positive value = improvement

VAS after test: visual analogue scale 0–100 mm (0 = no pain, 100 = worst imaginable pain), median and interquartile range, positive value = improvement

Borg CR 0–10 after test: neck muscle fatigue (0 = no fatigue, 10 = extremely strong fatigue), median and interquartile range, positive value = improvement

Deformation Sup.TR: deformation area upper part of trapezius, negative value = greater deformation area at 3 months follow-up

Deformation DeepTR deformation area lower part of trapezius, negative value = greater deformation area at 3 months follow-up

Table 2S. Correlations between change score in deformation area trapezius, neck pain, fatigue, neck disability and adherence to exercises

|  |  |  |  |  |  |  |  |
| --- | --- | --- | --- | --- | --- | --- | --- |
|  |  | **Upper trapezius** | **Lower trapezius** | **Neck pain (VAS)** | **Muscle fatigue (Borg)** | **Neck Disability (NDI)** | **Addherence exercise** |
| **Upper trapezius** | Correlation | 1 | -0.296 | 0.257 | ,446* | -0.332 | 0.07 |
|  | Sig. (2-tailed) | . | 0.61 | 0.226 | 0.029 | 0.122 | 0.75 |
| **Lower trapezius** | Correlation | -.296 | 1 | -0.306 | -0.106 | 0.095 | -0.171 |
|  | Sig. (2-tailed) | 0.161 | . | 0.146 | 0.62 | 0.665 | 0.434 |
| **Neck pain (VAS)** | Correlation | 0.257 | -0.306 | 1 | .474* | -0.289 | -0.232 |
|  | Sig. (2-tailed) | 0.226 | 0.146 | . | 0.014 | 0.161 | 0.265 |
| **Muscle fatigue (Borg)** | Correlation | .446* | -0.106 | .474* | 1 | -0.285 | -0.15 |
|  | Sig. (2-tailed) | 0.029 | 0.62 | 0.014 | . | 0.167 | 0.474 |
| **Neck Disability (NDI)** | Correlation | -0.332 | 0.095 | -0.289 | -0.285 | 1 | 0.066 |
|  | Sig. (2-tailed) | 0.122 | 0.665 | 0.161 | 0.167 | . | 0.744 |
| **Addherence exercise** | Correlation | 0.07 | -0.171 | -0.232 | -0.15 | 0.066 | 1 |
|  | Sig. (2-tailed) | 0.75 | 0.434 | 0.265 | 0.474 | 0.744 | . |
|  |  |  |  |  |  |  |  |
| * Correlation is significant at the 0.05 level (2-tailed). | | |  |  |  |  |  |

NDI: Neck Disability Index 0–100% (0% = no disability to 100 % = complete disability), median and interquartile range, positive value = improvement

VAS after test: visual analogue scale 0–100 mm (0 = no pain, 100 = worst imaginable pain), median and interquartile range, positive value = improvement

Borg CR 0–10 after test: neck muscle fatigue (0 = no fatigue, 10 = extremely strong fatigue), median and interquartile range, positive value = improvement

Deformation Sup.TR: deformation area upper part of trapezius, negative value = greater deformation area at 3 months follow-up

Deformation DeepTR deformation area lower part of trapezius, negative value = greater deformation area at 3 months follow-up
